# Practices and perspectives of primary care physicians in Japan and the United States about diagnosing dementia: a qualitative study

**DOI:** 10.1186/s12877-021-02457-7

**Published:** 2021-10-11

**Authors:** M. Abe, S. Tsunawaki, M. Dejonckheere, C. T. Cigolle, K. Phillips, E. B. Rubinstein, M. Matsuda, M. D. Fetters, M. Inoue

**Affiliations:** 1 Department of Family and Community Medicine, Hamamatsu University School of Medicine, Shizuoka Hamamatsu, Japan; 2 Omaezaki Family Medicine Center, Shizuoka Omaezaki, Japan; 3 Shizuoka Family Medicine Program, Shizuoka Hamamatsu, Japan; 4 Department of Family Medicine, University of Michigan, 1018 Fuller Street, Ann Arbor, MI 48104 USA; 5 Department of Internal Medicine, University of Michigan, Ann Arbor, MI USA; 6 VA Ann Arbor Healthcare System (VAAHS) Geriatric Research Education and Clinical Center (GRECC), Ann Arbor, MI USA; 7 Department of Sociology and Anthropology, North Dakota State University, Fargo, North Dakota USA; 8 Kikugawa Family Medicine Center, Shizuoka Kikugawa, Japan; 9 The School of Health Humanities, Peking University Health Science Center, Beijing, China

**Keywords:** Diagnosing dementia, Primary care physicians, Japan, United States, Qualitative comparison

## Abstract

**Background:**

While dementia is a common problem in Japan and the US, primary care physicians' practices and perspectives about diagnosing dementia in these different healthcare systems are unknown.

**Methods:**

Qualitative research was conducted in an ethnographic tradition using semi-structured interviews and thematic analysis in primary care settings across Japan and in the Midwest State of Michigan, US. Participants were a total of 48 primary care physicians, 24 each from Japan and the US participated. Both groups contained a mixture of geographic areas (rural/urban), gender, age, and years of experience as primary care physicians.

**Results:**

Participants in Japan and the US voiced similar practices for making the diagnosis of dementia and held similar views about the desired benefits of diagnosing dementia. Differences were found in attitudes about the appropriate timing of formally diagnosing dementia. Japanese physicians tended to make a formal diagnosis when problems that would benefit from long-term care services emerged for family members. US physicians were more proactive in diagnosing dementia in the early stages by screening for dementia in health check-ups and promoting advance directives when the patients were still capable of decision-making. Views about appropriate timing of diagnostic testing for dementia in the two systems reflect what medical or nursing care services physicians can use to support dementia patients and caregivers.

**Conclusions:**

Benefits of making the diagnosis included the need to activate the long-term care services in Japan and for early intervention and authoring advance directives in the US. Testing to establish an early diagnosis of dementia by primary care physicians only partly relates to testing and treatment options available. Benefits of making the diagnosis included the need to activate the long-term care services in Japan and for early intervention and authoring advance directives in the US.

**Supplementary Information:**

The online version contains supplementary material available at 10.1186/s12877-021-02457-7.

## Background

In 2015, about 47 million people were living with dementia globally and the number is projected to triple by 2050 [1]. Given the sheer volume of patients with dementia, primary care physicians have an increasingly important role in diagnosing and managing dementia in their daily practice [2–4]. While clear evidence for clinical utility of screening for dementia in primary care setting remains limited [5–7], many authors have suggested potential benefits of early diagnosis by arguing that therapeutic intervention could delay progression of dementia [1, 3, 8, 9], mobilize support that could help maintain quality of life for patients and their family members [3, 4, 10–12], and provide opportunity to discuss advance care planning or advance directives with dementia patients [10, 13–15].

However, there are many barriers in primary care settings to establishing the diagnosis of dementia early. Barriers noted include limited time for consultation while managing multiple chronic conditions [2, 3, 12], uncertainty about diagnostic resources including concerns about making diagnostic errors [3, 16], and limited effectiveness and the high cost of treatment [12, 16]. Additionally, concerns have included increasing the fear or stigma of patients and family members [2, 16], and lacking resources in the community for supporting dementia patients and family members [16, 17]. Weighing the benefits of an early diagnosis of dementia is a complex arena for primary care physicians trying to pursue a ‘best interests’ approach for patients and their care providers [16].

Previous research about diagnosing dementia in the primary care setting has largely been conducted in the United States (US), United Kingdom, and Australia [6]. Even though Japan is one of the most aged countries in the world and the social burden of dementia is growing [18, 19], few studies have described the practices and perspectives of Japanese primary care physicians on this issue. Previous scholarship has noted that dementia has different sociocultural contexts [20–22] and medical practice is affected by public health measures and local care resources [23–25]. Therefore, understanding facilitators or barriers for establishing a dementia diagnosis unique to Japanese primary care physicians [26] could be further contextualized by comparing practices in Japan with practices in a different setting [27].

The premise for this investigaton is that cross national research on establishing a diagnosis of dementia could enhance understanding of Japanese and US primary care physicians’ approaches to patient care. To contextualize this comparison, several factors should be considered. First, both countries are experiencing rapid aging in their populations. The share of the population over the age of 65 is 27.7% in Japan and 15.6% in the US [28–29]. Both countries have a large population of patients with dementia. There are an estimated 6.3 million persons in Japan and 5.8 million in the US with dementia even though the latter has more than twice the total population [30–32].

Second, the elderly in both countries have access to health insurance but with some important differences. Japan has offered universal health care coverage since 1961 [33]. Long-term care insurance was established in 2000 as a component of national social security to help the elderly with needs associated with accessing health care services [24, 31, 34]. In the US, there have been two primary types of public health insurance systems since 1965: Medicare for citizens of 65 years and older, and Medicaid for low-income citizens and other meeting certain disease criteria. Insurance coverage varies by individual depending on their past and present employment condition [35], and unlike Japan’s universal access, long-term care insurance in the US is very expensive and available through private insurers [36]. These differences aside, both countries have technophilic cultures with technology relatively easy to access. Likewise, both health insurance systems incentivize using health care technology [25].

Third, the role and characteristics of primary care physicians differ in the two countries. In Japan’s system, patients are allowed to visit any physician or multiple physicians without referral [37]. In contrast, patients typically require referral from their primary care doctors in the US system as an approach to help minimize health care costs [38]. In Japan, a community-based integrated care system was implemented by the government in 2012 to activate seamless healthcare services with a multidisciplinary approach for the elderly and people living with dementia [30, 39]. Primary care physicians play a central role in this plan, however a formally recognized system of family medicine is still new in Japan. Despite a much longer history in a handful of programs that launched family medicine training, systematic residency training programs to produce family physicians didn’t start growing until around 2007 [37, 40]. It wasn’t until 2017 that *sogoushinryou,* translated as general medicine, became the 19th specialty in Japan [37].

In Japan, the vast majority of physicians who were working as primary care physicians before establishment of the specialty in 2017 had trained first in hospitals as subspecialists before opening a private outpatient practice as a second career [41]. Arguably, they are hybrid subspecialist/primary care physicians based on their provision of a mix of subspecialty care learned during their hospital training, and first contact care for common acute and chronic conditions [42]. Like Japan, family physicians play an important role in communities as the most common primary care provider in the US. In contrast to Japan, family medicine in the US was recognized as a specialty nearly five decades earlier in 1969. In the US, primary care physicians are systematically trained to provide a broad range of care which prepares them to care for a wide range of health issues of patients of various ages in a continuous relationship [38, 41]. Due to a different system that encourages privileging for local hospital inpatient work, many family doctors, especially those in rural areas, practice a combination of inpatient care in community hospitals and local outpatient care, a source of continuity for hospitalized elderly.

Fourth, the two countries have very different patient and family participation in decision making and sharing of information [25, 43]. The US has implemented advance directives since the 1990s [44]. While perspectives on the effectiveness of this approach vary [13, 45], advance directives do help formalize surrogate medical decision making when an individual can no longer make decisions. In Japan, the concept of “advance care planning” was introduced in the late 2000s but uptake by physicians is still low [46]. In contrast to the US, the system of advanced care planning does not have legal protection for surrogate medical decision making in Japan [47].

Given these pertinent demographic and sociocultural similarities and differences, the purpose of this study was to explore the practices and perspectives of primary care physicians on the mutually common problem of diagnosing dementia in Japan and the US. By comparing the similarities and differences between the two countries, we aimed to understand the approach and challenges of primary care physicians’ for diagnosing dementia in each healthcare system.

## Methods

### Design

The present study utilized qualitative methods grounded in an ethnographic tradition that was needed due to the lack of descriptive research about primary care physicians’ management of dementia. It is a part of a larger investigation of a rural-urban comparison of primary care physicians’ dementia care for multimorbid older adults conducted in Japan and the US. Data collection by semi-structured interviews was conducted in Japan from August to October in 2017, and in the US between August 2015 and June 2018.

### Setting

In Japan, participants were recruited from physicians residing throughout the country for a national sample. In the US, participants were recruited from primary care physicians in the State of Michigan. Located in the Midwest, this single state has a broad range of industries, agriculture, racial and ethnic populations, as well as rural, suburban, and urban settings. Michigan has relatively comparable aging and dementia rates with the US overall and is relatively comparable with Japan as shown in Table 1.

**Table 1:** Population and dementia prevalence in Japan, US, and Michigan. (2020)

	Japan	U.S.	Michigan
Total population	126 million	327 million	10 million
Share of the population aged over 65 and older	27.7%	15.6%	17.7%
Population living with dementia, 65 and older	6.3 million	5.8 million	0.2 million
Dementia population rate among 65 and older	17.5%	11.4%	10.7%

Sources: OECD, 2020; OECD, 2019; United States Census Bureau, 2019; Ministry of Health Labour and Welfare, 2017; Alzheimer’s Association, 2020.

### Data collection instrument and reflexivity

Based on a review of the literature, and their extensive knowledge of the two country’s primary care systems, the team members created the interview guide. We used a semi-structured interview format which allowed the interviewer to ask additional questions of interest as they emerged [48].

Interview questions addressed primary care physicians’ goals in managing multimorbid patients with dementia; their practices of diagnosing, disclosing, and managing dementia; their comfort level with these tasks; and available resources for dementia care in their working environment (Supplement 1). We collected trajectories of several memorable cases from each participant and probed about their perspectives and approaches of diagnosing dementia. The investigators developed the interview guide first in English, and then translated it for use in Japan.

Four team members were assigned as interviewers which included a qualitative research methodologist with training in intercultural communication (MA) in Japan, and in the US, a geriatric pharmacist (KP), an anthropologist (ER), and a medical and public health student. All interviewers were trained to conduct qualitative interviews and had no relation with the participants in advance.

### Recruitment, sampling and data collection procedures

The goal of our purposeful sampling was to recruit in both countries a mixture of physicians based on rural/suburban/urban practice location, gender, age, and years of experience as a primary care physician in accordance with maximum variation sampling. In Japan, participants in the research were members of Japan Primary Care Association and who had received training as a family physician. Specifically, we sought representation from geographical areas in the north and south regions of the country including remote islands. The details of the recruiting process have been described elsewhere [26]. In the US, recruitment began by approaching primary care physicians including family physicians and internists practicing within the Great Lakes Research Into Practice Network (GRIN) [49]. Information regarding the study was distributed to physicians in the network, and those with an interest in participating were contacted to confirm eligibility. To expand access to practitioners’ experiences and clinical backgrounds, we expanded our sampling to include primary care physicians from rural and metropolitan areas outside the GRIN network.

Interviews were conducted in person at physicians’ offices, telephone/voice over the internet, or using video conferencing. Interviews lasted 60 to 150 min in Japan and 30 to 90 min in the US. All were audio-recorded and transcribed verbatim. Although the dementia care process was discussed broadly during the interviews in both locations, this paper focuses on primary care physicians’ practices and perspectives about diagnosing dementia.

### Ethical considerations

We informed participants about the voluntary nature of study participation and the freedom to withdraw from the study. Human subjects review and ethical approval was obtained from The Institutional Review Board of Hamamatsu University School of Medicine in Japan [No.16–233] and Health and Sciences and Behavioral Sciences Institutional Review Board of the University of Michigan in the US [IRB: 00000246].

### Qualitative data analysis

After each interview, the interviewer created field notes, using the “3C’s approach” [50] addressing the three constructs of “context”, “content” and “concepts” to capture the major issues that were discussed during the interview. The Japan team members translated their 3C’s summaries into English to promote exchange of the results with the US team several times over the course of data collection. Each team proceeded with a thematic analysis to the transcribed interview data in their language which facilitated an understanding of the perspectives of physicians in their own words. MAXQDA Analytic pro 12 was used for data coding.

For the Japanese data analysis, the research team (MI, ST, MM, MA) regularly shared the 3C’s summaries in scheduled research meetings to discuss the key conceptual ideas from each interview. To allow for a naturalistic process not encumbered by language and cultural differences based on the US format, the initial coding scheme in Japan created by the analyst (MA) contained the study’s major constructs, e.g., diagnosis, disclosure, management, the end-of-life care, and sub-codes were added in response to emerging findings from the team discussions. For the US data, after the first eight interviews were conducted, the research team began developing a coding scheme. Then three researchers (CC, KP, MD) applied the codes to the first eight transcripts and modified the coding scheme by adding additional codes that were not yet reflected. The remaining transcripts were coded by two analysts (MD, ER) and discussed by all team members. After conducting 24 interviews by each team, the research team members agreed that thematic saturation had been reached in both samples. The 3C’s summaries were reviewed during data analysis.

To conduct member checking, a process of sharing the overall findings with participants [51], we distributed a summary of the project findings, a copy of each participants’ interview summary, and a request for feedback. In Japan, all 24 participants replied with an agreement with the distributed summary, and 22 participants provided additional comments that we incorporated into the findings. In the US, the 24 participants were emailed information about the study findings though one email did not function. The 9 responding participants all indicated agreement and added a few clarifications that we incorporated into the findings.

### Japan-US comparisons

The lead analyst from Japan (MA) visited the US to compare the data coding and analysis approach with the US team members (MF, MD, CC, KP). This ensured that the coding schemes equivalently functioned for examining constructs of mutual interest. Later in the process, the lead US analyst (MD) visited Japan for a focused discussion of the present study. We first compared the coding scheme between Japan and the US under the category of “diagnosis” and related themes. Because the US had a more detailed scheme, MA reviewed Japan data and conducted additional coding while referencing the US scheme. There were several codes that emerged only from the Japan or the US data. MA and MD discussed the differences and set up several comparable themes such as primary care physicians’ practices of diagnosing dementia, cooperation with specialist physicians, perceived benefits of dementia diagnosis in primary care, and appropriate timing of diagnosing dementia in the views of primary care physicians. For each of these themes, MA first drafted a descriptive summary from Japan data and selected several quotations that represented the findings while MD did the same with the US data. Finally, we compared both descriptive summaries and discussed the similarities and differences and factors that affect the approaches of diagnosing dementia for the participating primary care physicians. The main codes and sub-codes related to “dementia diagnosis” are presented in Table 2.

**Table 2:** Coding scheme of perspectives of primary care physicians on diagnosing dementia (2017)

Main Codes	Sub-codes
Timing of diagnosis	
Confidence in diagnosis	High confidence
	Low confidence
Diagnostic process	Annual exams
	MD observations/knowledge
	Patient self-reports symptoms
	Family reports symptoms
	Clinical staffs/neighbors report symptoms *
	Understanding the patient’s life history/living environment *
	Taking clinical history from family/patient †
	Seeing only advanced cases
	Diagnostic ambiguity
	Assessments & tests
	Collective decision-making with family *
Confirming diagnosis through specialist	For better diagnostic tools
	For specialist resources
	Because diagnosis is unclear
	For acceptance of family/patient
	To plan treatment/help with treatment
	Lack of access to specialists
	Lack of access to neurocognitive testing
	Because it’s too hard for primary care physician to handle
Benefits of diagnosis	Safety
	Planning for the future
	Long-term insurance
	Access to care resources
	Knowing/acceptance of diagnosis by patient/family
	Acceptance/stigma in the community
	Treatment planning

*code only used in Japan

†code only used in the US

## Results

A total of 48 primary care physicians, 24 each from Japan and the US participated in the interviews and their profiles are illustrated in Table 3. In Japan, physicians based in local hospitals that typically have active outpatient departments were included because they were actively providing primary care services. Five participants had experiences of working in both urban and rural areas. In these cases, we asked them to speak primarily about their experiences in the environment leaving the strongest impression on them and to compare the two environments whenever appropriate. In the US, all physicians (100%) practiced in clinics. Because Japan is much more densely populated than the US, we used participants’ own categorization of their practice setting as suburban versus rural.

**Table 3:** Characteristics of participants (*n* = 48)

	Japan (*n* = 24)		US (*n* = 24)	
	Rural	Urban	Rural	Urban
	(*n* = 12)	(*n* = 12)	(*n* = 12)	(*n* = 12)
Years practicing as physician ^a^	12.7 ± 5.9	17.4 ± 8.1	7.9 ± 6.6	19.3 ± 9.0
	(6–24)	(9–38)	(2–23)	(6–34)
Gender^b^				
Male	9 (75%)	8 (67%)	5 (42%)	5 (42%)
Female	3 (25%)	4 (33%)	7 (58%)	7 (58%)
Setting^b^				
Clinics	8 (67%)	10 (83%)	12 (100%)	12 (100%)
Hospital	4 (33%)	2 (17%)	none	none

^a^Average ± SD, ^b^numbers(%)

### Practices of diagnosing dementia

We found that primary care physicians in Japan and the US used a similar approach to diagnosing dementia. Physicians commonly administered a screening test that most typically among these participants was the Mini-Mental State Examination (MMSE). They reported conducting blood work to rule out treatable diseases, checking medications to ensure impaired cognition was not a medical side effect, examining for underlying depression or mental illness, and considering use of a brain MRI or CT scan. They reported referring patients occasionally to a non-primary care specialist doctor for more testing to determine a specific type of dementia. In addition to above actions, these physicians emphasized speaking with patients’ family members or caregivers to take additional history and to understand the patients’ conditions at home. Many felt this information was equally or often times more important than doing other tests for diagnosing dementia.

There were minor differences in the screening tools used in Japan and the US. While the Mini-Mental State Examination (MMSE) was most common in both countries, participants in Japan also reported using the Hasegawa’s Dementia Scale (HSD-R) while those in the US used the Montreal Cognitive Assessment (MoCA) and Clock-Drawing test as alternatives.

Attitudes regarding specialist referral varied by the individual. Some participants reported always sending patients to a specialist for a formal diagnosis of dementia while others reported basically making the final diagnosis by themselves except in cases where they felt the need for more detailed testing. Additionally, participants in both countries were influenced whether or not to refer to a dementia specialist according to the wishes of patients’ family members.

*I may ask for a specialist’s advice if there are some concerns. And if the patient’s family members are hoping to see a specialist then I will introduce them. Other than that basically, I keep taking care of them. (Japan_Urban_24)*

*I would say if the Montreal Cognitive Assessment (MoCA) Test is really abnormal, and I feel confident, based on the symptoms, and the caregiver, and all that stuff, I’m going to diagnose. With or without imaging. (US_Urban_M04)*

We asked participants about their confidence in making the diagnosis of dementia. Most of them were confident about their knowledge of the procedure for making the diagnosis given their access to specialists in circumstances if they were not comfortable with the case. The level of confidence for making a dementia diagnosis was markedly higher for obvious cases of dementia, for patients with advanced age and cognitive symptoms, and especially when family members could provide a compelling history for the patient. In contrast, situations that lowered the physicians’ confidence in making the diagnosis included an early stage of dementia, suspicion of dementia in younger patients, lacking access to family members to discuss the situation or when patients lived by themselves, and a clinical setting which did not have good access to specialists or imaging tests. Varying levels of confidence were consistent between physicians in Japan and the U.S.

The lack of access to specialists was noted particularly in rural areas in both Japan and the US but for different reasons. In Japan, particularly in remote islands, concern focused on the burden on patients of traveling to a larger hospital outside the island. In the US, the concern was both distance and a long waiting time for an appointment with a specialist.

*(In urban area) you can do imaging and blood tests routinely to make a diagnosis but in the environment, where we could not easily access testing, I was less comfortable in making a diagnosis of dementia when in a rural area. (Japan_Rural and Urban_17)*

*I’ll usually send them out to the neuropsychiatric testing, and I’ve had people very willing to pay for that, although you wait a long, maybe six months, to get it scheduled and actually have it completed. (US_Urban_M03)*

### Perceived benefits of establishing the dementia diagnosis in primary care

Due to the perception that dementia is not curable even with the latest treatment options, treatment was not the main purpose of diagnosing dementia for primary care physicians. The benefits of diagnosing that they emphasized were: ensuring the safety of patients and the community, gaining access to additional care resources, making future plans, and improving the well-being of patients and their family members.

The formal establishment of the diagnosis of dementia was found beneficial for gaining access to appropriate care services and insurance eligibility. Primary care physicians in Japan and the US reported supporting their patients and their family members by discussing the prognosis by providing guidance on what will happen in the immediate future for the dementia patient, advising how to prepare, discussing the kinds of care appropriate for the patient. Planning places to live/die emerged as a major topic to be considered in the long-term.

*If they can use long-term care services, there are various day service facilities. For instance, just to spend the daytime and come home, or those multi-functional type facilities that are very useful where the same staff provide home care and accept short-term stays in the facility. (Japan_Rural_23)*

*Patients having that diagnosis open the door up to other services that we can get, whether it be home health care, certain in-home devices, some medication administrative assistance. Even long-term care in extended-care facilities. (US_Rural_R11)*

Medications that are commonly used in Japan were donepezil, memantine, galantamine, and in the US were donepezil and memantine. However, many participants said that they did not see a clear effect of these medications for dementia. Even though both Japanese and the US participants explained to patients and their family members that medication could potentially slow down the progress of dementia, they did not impose medications on patients if they did not wish to take them.

*There are those who want to take medication if available, and there are those who don’t want to if the medications aren’t so effective. It all depends, so I try to make sure to ask his/her preference at the beginning. (Japan_Urban_19)*

*I have very mixed feelings about even starting people on the medications because I feel like the evidence for them that they are effective is pretty limited. (US_Urban_U06)*

There were participants from both countries who stated that they didn’t see much value in specifying the type of dementia or making fine adjustments in dementia medications according to diagnoses. Such ideas were shared when contrasting their own practice to the typical approach of dementia specialists. As primary care physicians, they focused more on the mental care and life support for patients and care givers in the belief of pursuing holistic care by balancing the benefits of available treatment options.

### Diverse views on the timing of diagnosing dementia

Primary care physicians had knowledge for establishing a dementia diagnosis and knew the benefits of diagnosing dementia. However, how and when to bring up the topic with patients could be a sensitive issue. Participants in Japan and the US shared similar recognition of features related to diagnosing dementia such as cognitive function usually declining slowly, that there could be diagnostic ambiguity, that there was social stigma associated with the disease, and a hesitancy to ‘label’ the patient.

In our probing about participant’s memorable cases and their trajectories from the diagnosis to final treatment, the common triggers of suspecting dementia in patients were family members reporting symptoms and patients not taking medications. However, we observed different trends in the process of suspecting and determining dementia diagnosis in Japanese and the US participants. In Japan, primary care physicians described involvement of a wide range of individuals beyond the family members for detecting dementia. For instance, receptionists or nurses may express concerns about patients based on their behavior outside examination rooms. There were reports of caseworkers or neighbors who were worried about patients’ living conditions and sought help from clinics. Some Japanese physicians were building community networks by routinely attending meetings with agencies that managed security in the area such as fire and police departments.

*I rarely had noticed the first signs of dementia. In the majority of cases, I understood for the first time after listening carefully to other people’s opinions which suggested that patients possibly had dementia. (Japan_Rural_07)*

In many cases that the Japanese participants discussed, the doctor-patient relationship with a dementia patient often had started when there was suspicion that the patient had dementia. Another typical pattern occurred when a physician took over the responsibility of a patient’s care towards the end of life, e.g., when the patient switched to home care and the patient was already diagnosed with dementia at the time.

*Her dementia was already in advanced stage when I first saw her. She had been in a facility, but her daughter wanted to take care of this patient at home for the remaining time and asked me if I could provide home visits for them. (Japan_Urban_2)*

In contrast, commonly, US primary care physicians reported having had a relationship with their patients from an earlier stage of their lives and were able to notice changes associated with dementia during wellness visits. It was observed that the process is often nuanced and slow to evolve. The fact that dementia screening was required in the annual wellness visits for patients who were covered on Medicare prompted physicians to be on the watch for early signs of cognitive decline.

*I’m starting to get from actually our Medicare Annual Wellness Visit Form, which has a question that will say something about memory, and they’ll circle, “Yes,” just really not thinking anything of it. And then we start to have that conversation. (US_Urban_M04)*

Only one physician in Japan reported screening for dementia as a regular part of a health check. In many cases Japanese physicians took a ‘wait and see approach’ until it became evident that patients’ family members or neighbors were having problems. Under the long-term care insurance system in Japan, a practical benefit of diagnosing dementia was to meet eligibility criteria for services and to access benefits under the system. The long-term care insurance covers the involvement of both patient care coordinators called “care managers” and visiting nurses to support patients’ lives including end-of-life care. The link between establishing the diagnosis of dementia and accessing long-term care services in practice was confirmed by reports of some physicians who viewed diagnosing dementia not as particularly meaningful on isolated islands where resources for public care services were limited and where family members and neighbors provided supportive care for the elderly.

*I’ve been asked quite a few times to write documents that were required in order for patients to activate the long-term care services. (Japan_Urban and Rural_05)*

*The long-term care insurance does not offer that many services anyway, so I did not used to write that many letters indicating the diagnosis (of dementia on an island). (Japan_Rural, remote island_14)*

Additionally, another trigger for US participants to make an early diagnosis was reported as coming from systematic prompting and incentives to have patients complete advance directives. In completing an advance directive, patients are able to legally name a surrogate decision-maker who could make decisions regarding medical procedures on behalf of patients who are no longer able to do it for themselves. Participants shared the belief that this was a way to secure a future consistent with the patient’s preferences and that prompting for communication about this topic was built into the annual wellness visit.

*We always want the patient to name someone who can make decisions for them. We do something like advance planning built into our screening process --- all patients actually. (US_Urban_U01)*

In contrast, Japanese primary care physicians often described the process of discussing housing and end-of-life care issues with families of patients with advanced dementia, but decision-making systems such as the advance directive were not mentioned as a motivator for early diagnoses of dementia.

**Fig. 1:**
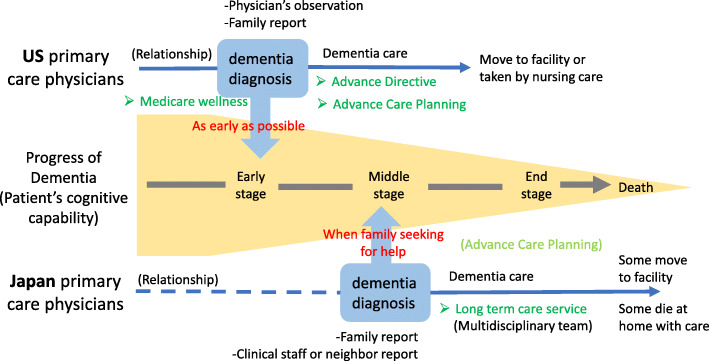
Conceptual model of primary care physicians’ approaches to diagnosing dementia and factors affected

Figure 1 serves as a conceptual model of diagnosing dementia by Japanese and the US primary care physicians based on the identified factors. The diagram depicts a central arrow demarcating the progressive nature of a patient’s dementia and cognitive decline from early stage until death. The bottom illustrates an overarching timeline of events for the trajectory in Japan and the top is an overarching timeline of events for the trajectory in the US. In the diagram, “relationship” refers to the time of the physician being responsible for the patient’s health care management. Many of the US primary care physicians had been seeing their dementia patients even before symptoms appeared, whereas participating Japanese physicians were more likely to have been consulted after family members identified concern about dementia. The social systems which frame the timing of diagnostic intervention of dementia were “long-term care insurance” in Japan and “advance directives” in the US. The concept of “advance care planning” was pervasive in the US. However, in Japan, advanced care planning was not raised as a motivation for early diagnosis but was sometimes mentioned in the context of end-of-life care.

## Discussion

By comparing the experiences of 48 primary care physicians, 24 each in Japan and the US, we have identified similarities and differences in their practices and perspectives about diagnosing dementia. Similarities were their clinical practices for establishing a diagnosis of dementia, views on potential benefits to patients and family members of diagnosing dementia, and common values prioritizing the quality of life and well-being of patients and caregivers rather than a focus on the diagnosis or medical treatment of dementia. This reflects a widely advocated role for primary care doctors to provide patient-centered care in the context of long-term relationships with patients and their family members [3, 4, 52].

The most striking differences between Japan and the US primary care physicians were perceptions about the appropriate timing and behaviors relative to diagnosing dementia. The US physicians were more proactive in diagnosing dementia in the early stages, while Japanese physicians tended to make the diagnosis when family members were encountering apparent problems. This difference seems to be influenced by their respective social systems available for supporting dementia patients. The following three factors are identifiable reasons: national strategies for early detection of dementia, the linkage of dementia diagnosis and care support, advance directives, to understand primary care physicians’ approaches and challenges.

First, regarding national strategies for dementia care, both Japan and the US emphasize early detection of dementia, but the division of responsibility for its implementation takes a different form. In the US, there is an official recommendation in medical settings to provide “a simple cognitive test during the annual Medicare wellness visit” [53, 54]. The wellness visits are regularly made by primary care physicians [32], so the entry point for medical detection on dementia is clear. Whereas in Japan the first access point of dementia care pathway is ambiguous and undetermined [24]. Since 2015, the Japanese government is promoting the “New Orange Plan” strategy to strengthen the development of dementia-friendly communities to prepare for the rapid increase of people with dementia. The 7 pillars of the Plan includes the operation of “initial intensive support team for dementia” which consists of a group of doctors, nurses, public health nurses, and care managers [55, 56]. The plan focuses on a team approach to find and help people who potentially have dementia within the local community unit. Screening for dementia is to be conducted as part of public care-prevention service by each local government [30,57], but the implementation rate remains considerably low [58]. Japan does not have a general practitioner system for elderly patients with chronic illnesses to visit regularly. Instead, it is common for patients to visit multiple private practice subspecialists according to the patient’s medical diagnoses. As a result, ambiguity of responsibility is prevalent in prevention or early detection of dementia in primary care services in Japan as subspecialists are not trained to coordinate care as would a family physician. This explains partially the differences we observed in our study.

Second, access to care support was considered a major benefit of making the dementia diagnosis in both countries. However, it was more clearly recognized in Japan as affordable services are more available. The diagnosis of dementia by primary care physicians was significantly linked to motivation to utilize long-term care services for patients and support family caregivers. This indicates that the long-term care services for the elderly have become widely used since the enforcement of the Long-Term Care Insurance Act in 2000 in Japan [30, 59]. Under the insurance system, there are multiple care service providers in place that facilitate utilizing comprehensive services under the medical diagnosis of dementia [31]. In this regard, we observed that in the US, patients’ access to care support varied depending on the physician’s practice environment, and a diagnosis of dementia was not always associated with enhanced care provision. This may be a challenge for dementia care in US primary care [3].

Third, the presence of an advance directive system could affect primary care physicians’ attitudes about early intervention for dementia. Among US primary care physicians, communication about advance directives has been incorporated in the patients’ wellness visit, and patients are asked routinely to appoint a surrogate decision-maker. However, in the current Japanese adult guardianship system, a proxy can be appointed only for property management and not medical decision-making [47]. Medical and nursing care plans for the elderly are often decided collectively by family members and medical and nursing professionals, considering what the patient would have wanted in that situation [25, 26, 60]. While efforts promoting advance care planning in Japan began in 2007 [46], uptake has been slow [61, 62] and does not include any legal directives.

But many agree that advance care planning as an approach enabling the elderly to prepare for the final stage of their lives by explicitly stating their preferences will gradually expand in the future [46, 61, 63]. In a survey of the Japanese public’s attitude toward dementia diagnosis and disclosure [64, 65], the majority preferred to know the diagnosis as soon as it was made. The reasons for this were that they would like “to access treatment and support” and “to consult or convey their will for the future.” Intergenerational comparison showed that the younger generation tended to be more proactive in preparing for their future risks [64]. Such results suggest that if the patients’ preferences discussed through advance care planning will become legally protected in Japan, medical professionals may feel more compelled to communicate with those suspected of having dementia at an earlier stage of the disease.

What can be inferred from the above discussion is that the concept of appropriate timing of diagnostic intervention for dementia reflects what medical or nursing care services physicians have available to them. The lack of testing and curative medication, and the social stigma associated with dementia serve as barriers to formally make the diagnosis and render disclosure difficult [26]. However, primary care physicians will intervene when they find benefits that outweigh these barriers. Therefore, not only the availability of early diagnostic measures such as biomarkers [66], but also the availability of diagnosing systems and care services in place for patients that can be used after diagnosis is necessary to promote early diagnosis and advance care planning initiatives.

A strength of the present study is the collection of practices and perspectives of primary care physicians about diagnosing dementia both in Japan and the US. By listening to various trajectories of long-term dementia care, we were also able to gain insights into socio-cultural aspects of diagnosing dementia [20–22] and understanding physicians’ intention behind their practices in depth. Moreover, by comparing the practices in two countries with different health care systems and cultural backgrounds, with a multicultural research team we were able to explore the characteristics of both countries that may not be easily recognized by examining one system in isolation [20, 23]. Comparative studies of countries such as this can be used to examine different methods and systems beyond existing systematic boundaries [27].

A potential limitation, or at least a particular challenge of cross-national research, especially in a qualitative study, involves the selection of comparable participants. In the present study, participants were predominantly recruited from the membership of the Japan Primary Care Association, while in the case of the US, they were all primary care physicians in the state of Michigan. We do feel the findings are transferrable in both Japan, and for the Michigan sample to the broader US. These findings identify the kinds of challenges and approaches characteristic in each country. Additionally, it is plausible that the interviewers’ differences in backgrounds could have impacted the information drawn from the participants in the two countries since the interviewer is the "instrument of data collection" [48]. Still, in spite of geographic, language, and sampling strategy challenges, we communicated about the process throughout the study and pursued a rigorous approach.

## Conclusion

The challenges faced and strategies used by Japan and US primary care physicians are remarkably similar relative to diagnosing dementia. That said, there are differences. The US primary care physicians were more proactive in diagnosing dementia at an early stage, while Japanese counterparts tended to wait until the problems faced by the family members became apparent. Efforts promoting the early diagnosis of dementia need consider not only the testing and treatment measures available, but also the long-term care services and an advance directive system that makes establishing the diagnosis clearly beneficial to patients.

## Supplementary Information

**Additional file 1.** Interview Guide.

## Data Availability

The datasets used and/or analyzed during the current study are available from the corresponding author on reasonable request.

## Abbreviations

US: United States
GRIN: Great Lakes Research Into Practice Network
MMSE: Mini-Mental State Examination
HSD-R: Hasegawa’s Dementia Scale
MoCA: Montreal Cognitive Assessment
